# Of Mice and Men — Universality and Breakdown of Behavioral Organization

**DOI:** 10.1371/journal.pone.0002050

**Published:** 2008-04-30

**Authors:** Toru Nakamura, Toru Takumi, Atsuko Takano, Naoko Aoyagi, Kazuhiro Yoshiuchi, Zbigniew R. Struzik, Yoshiharu Yamamoto

**Affiliations:** 1 The Center for Advanced Medical Engineering and Informatics, Osaka University, Osaka, Japan; 2 Osaka Bioscience Institute, Osaka, Japan; 3 Educational Physiology Laboratory, Graduate School of Education, University of Tokyo, Tokyo, Japan; 4 Department of Psychosomatic Medicine, Graduate School of Medicine, University of Tokyo, Tokyo, Japan; University of Cambridge, United Kingdom

## Abstract

Mental or cognitive brain functions, and the effect on them of abnormal psychiatric diseases, are difficult to approach through molecular biological techniques due to the lack of appropriate assay systems with objective measures. We therefore study laws of behavioral organization, specifically how resting and active periods are interwoven throughout daily life, using objective criteria, and first discover that identical laws hold both for healthy humans subject to the full complexity of daily life, and wild-type mice subject to maximum environmental constraints. We find that active period durations with physical activity counts successively above a predefined threshold, when rescaled with individual means, follow a universal stretched exponential (gamma-type) cumulative distribution, while resting period durations below the threshold obey a universal power-law cumulative distribution with identical parameter values for both of the mammalian species. Further, by analyzing the behavioral organization of mice with a circadian clock gene (*Period2*) eliminated, and humans suffering from major depressive disorders, we find significantly lower parameter values (power-law scaling exponents) for the resting period durations in both these cases. Such a universality and breakdown of the behavioral organization of mice and humans, revealed through objective measures, is expected to facilitate the understanding of the molecular basis of the pathophysiology of neurobehavioral diseases, including depression, and lay the foundations for formulating a range of neuropsychiatric behavioral disorder models.

## Introduction

The quest for a principle underlying human behavior is considered to be a difficult task, since actions are subject to the individual's constant conscious deliberation and psyche, resulting in a continuously changing type and level of activity, arising from interaction with dynamically changing environmental demands. Yet, recent studies by Barabási et al. [1], [2] have pointed towards the existence of a universal statistical law governing waiting times of inter-human communication, such as e-mail exchange, Web browsing and trade transactions. Very recently, Nakamura et al. [3] also studied the nature of human behavioral organization, specifically how resting and active periods are interwoven throughout daily life, and found that the duration statistics exhibit universal behavior, which can be generalized across individuals.

In the present study, we show that in experiments wild-type mice with minimal environmental demands display exactly the same behavioral organization as healthy humans, suggestive of the presence of an underlying principle governing behavioral organization across these mammalian species. Surprisingly, we further show *mPer*2*^Brdm^* ^1^ mice [4], with a deletion mutation in the mouse *Period2* gene, display the breakdown of such a universal statistical law for resting periods, just as observed in patients with major depressive disorders [3]. Considering the reported chronobiological abnormality in depression [5]–[7], our findings have potential implications for the development of a novel mouse model of depression.

## Results

### Distribution laws of resting and active periods

**Figure 1:**
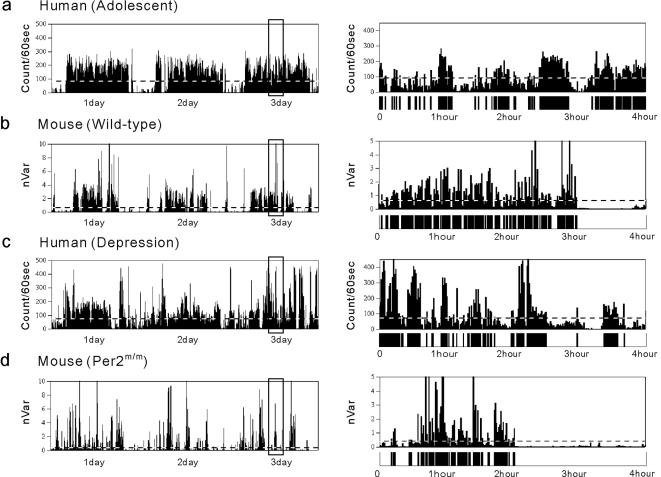
Fluctuation of locomotor activity in humans and mice. Illustrative examples of locomotor activity data for an adolescent (a), a wild-type (WT) mouse (b), a depression patient (c) [3], and a *Period2* mutant (*Per*2*^m^* ^/*m*^) mouse (d) over three consecutive days (left panels). The right panels are magnifications of the left panels with 4-hour periods during the 3rd subjective daytime. The overall average of non-zero activity counts is used for a threshold (horizontal dotted line), and the period in which the counts are successively below or above the threshold is coded respectively as a resting (open bar in bottom panels) or active (closed bar) period. Because the body weight varies across individuals, the mice's activity counts in the vertical axis (nVar) are normalized by the mean variance of each record.

**Figure 2:**
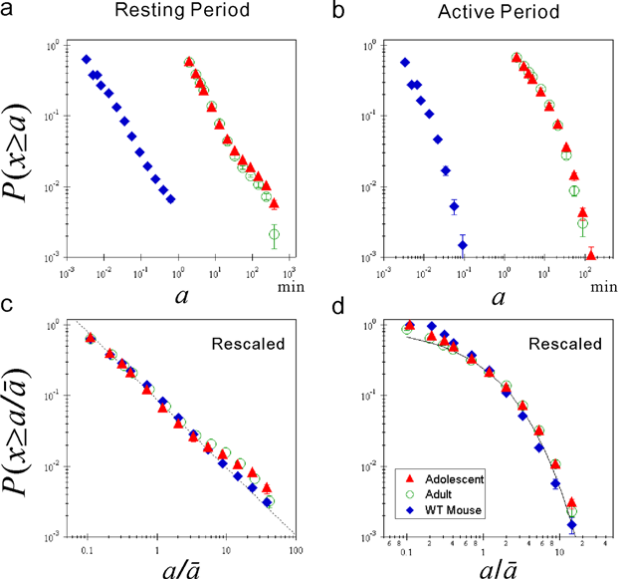
Universal distribution laws of behavioral organization. Cumulative distributions of resting and active period durations of locomotor activity in healthy humans and wild-type (WT) mice. (a) Cumulative distributions *P*(*x*≥*a*) of resting period durations *a* for human adolescents (closed triangles), adults (open circles) [3], and WT mice (filled diamonds). The error bars indicate the standard error of the mean. (b) The same as (a), but for active period durations. (c) Group averaged rescaled cumulative distributions of (a). The straight line is an eye-guide with the overall adult mean scaling exponents 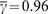 as the slope. Note that the plots are shifted along the vertical axis to take the same value at 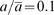 for the purpose of illustration. (d) The same as (c), but for active period durations. The solid curve is a stretched exponential function with the overall mean of values; 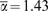 and 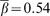.

Following Nakamura et al. [3] (see *Methods*), we analyze locomotor activity data, capturing even slight bodily acceleration counts in a continuous fashion (Fig. 1), and estimate the cumulative probability distribution *P*(*x*≥*a*) of durations *a* of both *resting* periods, where the activity counts are successively lower than a certain predefined threshold value, and of *active* periods, where the counts are successively higher than the threshold. When the overall average of non-zero counts is used as the threshold, the averaged cumulative distribution of resting period durations for 26 healthy human adolescents over on average 7 consecutive days (see *Methods*) take a power law form *P*(*x*≥*a*)∼*a* ^−*γ*^ over about 2 min to 100 min with the mean scaling exponent γ = 0.97±0.09 (mean±SD) (Fig. 2**a**). On the other hand, active period durations for healthy human adolescents obey a stretched exponential functional form *P*(*x*≥*a*) = exp(−*αa^β^*) rather than a power law distribution (Fig. 2**b**) with the mean *α* = 0.58±0.07 and *β* = 0.51±0.02 over about 5 min to 100 min. These distributions almost completely collapse onto those of the healthy adult group (*N* = 11) studied in our recent paper [3] (reproduced in Fig. 2 for comparison), indicating the robustness, universality and validity of the results.

### Universal distribution laws of behavioral organization

We conduct an analogical analysis of locomotor activity, measured by acceleration sensors placed under the cages (see *Methods*), of 12 wild-type mice over three consecutive days. Surprisingly, the averaged cumulative distribution of resting period durations of mice also obeys a scaling law for a wide range of durations *a* (from 0.1 sec to 60 sec) (Fig. 2**a**) with the mean scaling exponent *γ* = 0.95±0.08. Furthermore, the distributions of active period durations also follow a stretched exponential functional form (Fig. 2**b**). Note that the timescale taking a power law form is different from that for humans–more than 100 times shorter than for humans. Most importantly, the rescaled (by the individual mean duration 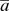) cumulative distributions (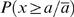) of resting period durations for healthy humans (adolescent and adult groups) and wild-type mice almost completely collapse onto the same power law distribution form (Fig. 2**c**) without significant differences in the rescaled exponents 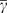 among groups (Table 1), in spite of the difference in the methods of data collection, species, weight, body size, lifestyle, etc. Moreover, the rescaled cumulative distributions of active period durations (Fig. 2**d**) well collapse onto the stretched exponential form for a wide range of timescales, without significant between-group differences in the rescaled parameters 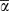 and 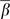 (Table 1).

**Table 1:** Fitting parameters of rescaled cumulative distributions.

	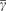	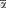	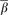
Adolescents	0.96±0.13	1.43±0.09	0.54±0.06
Adults	0.96±0.06	1.37±0.12	0.59±0.08
WT Mice	0.93±0.04	1.46±0.14	0.59±0.10
Human depression	0.78*±0.11	1.52±0.15	0.49†±0.07
*Per*2*^m^* ^/*m*^ Mice	0.84*±0.07	1.51±0.11	0.56±0.06

Values are mean±SD. *; *p*<0.05 from healthy adults and adolescents, and wild-type (WT) mice. †; *p*<0.05 from healthy adults and adolescents, and *Per*2*^m^* ^/*m*^ mice. The results for healthy adults and depression patients are reproduced from our recent study [3]. *Per*2*^m^* ^/*m*^ Mice: *Period2* mutant mice.

### Invariance of cumulative distribution with different threshold values

**Figure 3 pone-0002050-g003:**
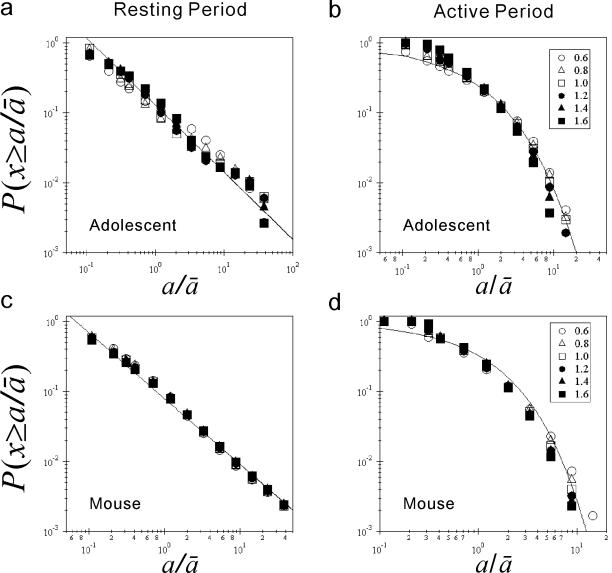
Invariance of the results with different threshold values. (a and b) Dependency of rescaled cumulative distribution on the threshold used to determine both resting and active periods in adolescents. (a) Rescaled (by the mean resting durations) cumulative distributions 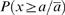 of resting period durations 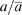, where 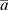 is the individual mean of the resting period durations, with different threshold values of 0.6, 0.8, 1.0, 1.2, 1.4, and 1.6 times the overall average for non-zero activity counts. (b) The same as (a), but for active period durations. (c and d) The same as (a), (b), but for wild-type mice. The straight lines in (a) and (c) are eye-guides with the mean scaling exponent from 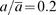 to 20 as the slope, with the threshold value of 1.0 (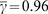 in (a) and 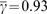 in (c)). The curves shown in (b) and (d) are stretched exponential functions *P*(*x*≥*a*) = exp(−*αa^β^*) with the mean 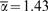 and 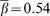 in (b) and 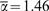 and 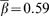 in (d), respectively.

The mean resting and active period durations are, by definition, influenced by the choice of threshold values; the higher the threshold, the longer the mean resting period and the shorter the active periods. We thus verify the invariance of the results with the threshold values used. Figure 3 shows the rescaled cumulative distribution with different threshold values ranging from 0.6 to 1.6 times the overall mean of non-zero activity counts. The cumulative distributions of both adolescents and wild-type mice collapse onto the power law form for resting period durations, and a stretched exponential function for active period durations, respectively, indicating that the *rescaled* cumulative distributions are virtually unaffected by choosing from a wide range of threshold values.

### Dependency of cumulative distribution on data resolutions

**Figure 4 pone-0002050-g004:**
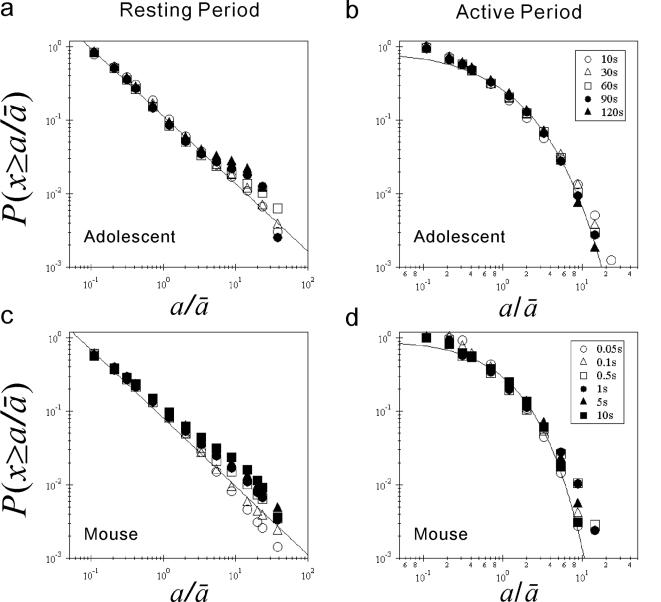
Dependency of cumulative distributions on data resolutions. (a) Rescaled cumulative distributions 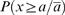 of resting period durations 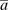 with different data resolutions of 10, 30, 60, 90, and 120 sec for adolescents. The error bars indicate the standard error of the mean. (b) The same as (a), but for active period durations. (c and d) The same as (a), (b), but for wild-type mice with different data resolutions of 0.05, 0.1, 0.5, 1, 5, and 10 sec. The straight lines in (a) and (c) are eye-guides with the mean scaling exponent from 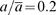 to 20 as the slope (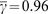 in (a) and 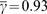 in (c)).

We also examine the influence of data resolutions, i.e., the length of the windows used to obtain activity counts, on cumulative distributions of both resting and active period durations. Figure 4**a, b** shows rescaled average cumulative distributions for the adolescent group with different data resolutions ranging from 10 to 120 sec. The cumulative distributions of resting period durations are not much altered in a range of scale from 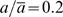 to 20. In the longer scales, the distributions gradually converge to the overall −1 slope (e.g. open circle) by increasing the data resolution. These results indicate the robustness of the scale exponents 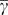, especially for the shorter scale [0.2, 20], against the data resolution. In addition, rescaled cumulative distributions of active period durations exhibit stronger robustness against data resolutions. In the case of wild-type mice, the rescaled cumulative distributions show a similar robustness against data resolutions ranging from 0.05 to 10 sec. Taking this into consideration, throughout this study, we chose a 1 min data resolution for humans and 0.1 sec for mice.

### Breakdown of universal distribution laws

**Figure 5:**
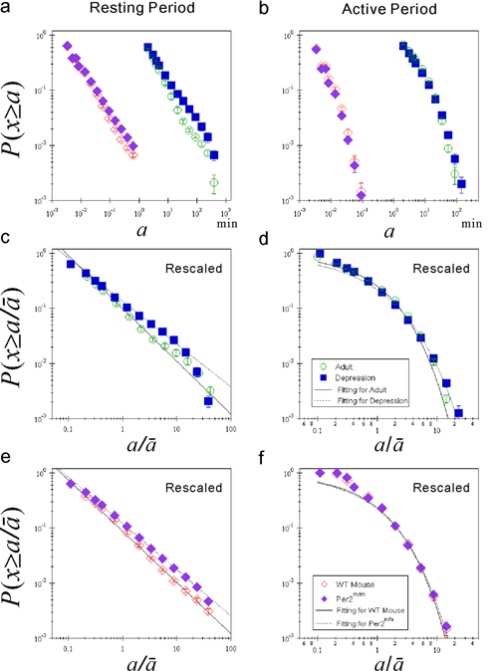
Breakdown of the universal distribution laws. (a) Cumulative distributions *P*(*x*≥*a*) of resting period durations for healthy human adults (open circles) and depression patients (closed squares) [3], wild-type (WT) mice (open diamonds) and the *Period2* mutant (*Per*2*^m^* ^/*m*^) mice (filled diamonds). The error bars indicate the standard error of the mean. (b) The same as (a), but for active period durations. (c) Group averaged rescaled cumulative distributions for healthy human adults and depression patients, reproduced from our recent study [3]. (d) The same as (c), but for active period durations. (e) Group averaged rescaled cumulative distributions for WT and *Per*2*^m^* ^/*m*^ mice. (f) The same as (e), but for active period durations. Note that the plots in (d) and (e) are shifted along the vertical axis to take the same value at 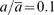 for the purpose of illustration. The lines are eye-guides with the group mean parameter values in Table 1.

Alteration in locomotor activity is one of the cardinal signs in psychiatric disorders, and many psychiatric disorders, including depression, indeed have diagnostic criteria which are evaluated through an assessment of altered locomotor activity [8]. For instance, one of the known disease signs in depression is *psychomotor retardation*, involving a recognizable alteration in locomotor activity, such as a slowing down of movement, which is observable by others [9]. In fact, in our recent study [3], we showed that the locomotor activity of patients with major depressive disorders (*N* = 16), in contrast to that of healthy controls, exhibits intermittent bursts in the activity counts with more episodes of slowing down or cessation of movement (reproduced in Fig. 1**c**), and the cumulative distribution of resting period durations departs from the universal power law observed for healthy controls, resulting in a significantly smaller mean scaling exponent 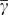 (reproduced in Fig. 5**a, c** and **Table 1**). In addition, these patients showed disrupted circadian rest-activity cycles, reflecting the reported chronobiological abnormality in depression [5]–[7].

Thus, we further study whether *mPer*2*^Brdm^* ^1^ mice [4] with a deletion mutation in the mouse *Period2* (*mPer2*) gene, a core clock gene, also having aberrant circadian rest-activity cycles (Fig. 1**d**) as often seen in human depression patients, display a similar breakdown of the universal statistical law for resting period durations. Nine homozygotes (*mPer*2*^m^* ^/*m*^) were studied over three consecutive days for their locomotor activity, just as with the wild-type mice. Very surprisingly, similar to human depression patients, the locomotor activity of these mutant mice exhibits bursts in the activity counts with more episodes of cessation (or slowing down) of movement (Fig. 1**d**). While the rescaled cumulative distributions of active period durations (Fig. 5**f**) well collapse onto the stretched exponential form observed for wild-type mice for a wide range of timescales, the averaged cumulative distribution of resting period durations of these mice consistently deviates from that of wild-type mice, just as that of human depression patients does (Fig. 5**a**). The rescaled cumulative distribution of resting period durations display a similar breakdown of the universal statistical law (Fig. 5**e**) to that observed in human depression patients (Fig. 5**c**), resulting in significantly smaller rescaled exponents 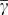 compared to both healthy humans and wild-type mice (Table 1). Therefore, we conclude that the *mPer2* mutant mice share the breakdown of the universal organization of resting periods with human depression patients.

## Discussion

Universality in waiting time distributions is abundant in a wide range of complex systems in nature, e.g. earthquake occurrences [10], [11], human communication [1], [2], and in neuronal avalanches [12]–[14], where it has been associated with critical branching processes [15], [16], and in systems showing (on-off) intermittent dynamics [17]. The existence of classes of invariant functional forms across scales, individuals, and even systems and their models, has played an important role in the quest for principles underlying such complex phenomena [18]. In this sense, it is particularly intriguing to observe the universal power law in the resting period durations of both humans and mice, because it belongs to an analogical class of universal statistical law in avalanche propagation durations experimentally determined in living neural networks [12]–successfully modeled by a critical branching process [16], and in the waiting time distribution of inter-human communication and its priority/motivation based model of its execution under random internal/environmental demands [1], [2]. Although an exact mechanism for our observation is yet to be determined [3], such an analogy strongly suggests the presence of an underlying neuro- and/or psycho-biological principle governing *healthy* behavioral organization, in particular onsets of spontaneous activity. In addition, the universality and robustness in behavioral organization found in both healthy humans and wild-type mice provides a window through which we can investigate its breakdown and which has molecular neuropsychiatric implications, as shown in this paper.

The human genome project has provided tremendous resources for medical and biological research, and medicine has become one of central fields in biological study. However, mental or cognitive brain functions and their abnormal psychiatric diseases, including depression, are still difficult to approach through molecular biological techniques, which are usually used as a powerful strategy in other biological areas. This is partly because no appropriate assay systems have been established in this field. Further, mental illnesses are heterogeneous and no objective examination methods for their diagnoses, such as blood tests or brain imaging, are used clinically even in humans. The establishment of animal models for neuropsychiatric diseases is confidently expected to facilitate the understanding of their molecular pathophysiology. We believe that the universal statistical laws of human and mice behavior, with the parameters characterizing the *normal* behavioral organization, and the simultaneous breakdown in *abnormal* individuals with depressive signs discovered in this study points towards a novel quantitative strategy that can be used in molecular neuropsychiatry, particularly for the development of animal models. To examine whether the parameters (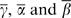) of the universal behavioral organization can be altered in other neuropsychiatric behavioral disorders would be an important next step.

## Materials and Methods

### Locomotor activity data of healthy adolescents

Locomotor activity data, defined as counts of events in which an acceleration signal crosses zero-level within a predefined time, were acquired from 26 adolescent subjects (junior high school students; 2 males, 24 females, 14±0.5 years of age) over on average 7 consecutive days (9.5±0.6 days, ranging from 5 to 10 days). All the participants wore a small watch-type activity monitor equipped with a computer (Ruputer Pro, Seiko Instruments, Chiba) [19], on the wrist of their non-dominant hand. This activity monitoring device is analogous in performance [20] to the commercial Actigraph Mini-Motionlogger (Ambulatory Monitors Inc., Ardsley, NY) [8], which is widely used in clinical fields, having the capability of detecting small changes in wrist acceleration (up to 0.01 *g*) so that even slight movements of the subjects are registered. Zero-crossing counts were accumulated for every 10 sec. The participants were instructed to wear the activity monitor at all times, except while bathing or during rigorous exercise such as physical education classes. They were also requested to record the times when they took off the device, went to bed and woke up. The periods they took off the device were not included in the analysis. Written consent was obtained from all the participants and their parents, and the study was approved by the research ethics committee of the Graduate School of Education, The University of Tokyo, to which the junior high school is attached. The methods of data collection and analysis are essentially the same as those used for the healthy adult population and for depression patients in our recent study [3].

### Locomotor activity data of mice

*mPer*2*^Brdm^* ^1^ mice (C57BL/6-Tyr^C-brd^) [4] were obtained from the Jackson Laboratory. Activity data were acquired from 12 wild-type mice (Male C57BL6/J) and nine *mPer*2*^Brdm^* ^1^ mice over three consecutive days. They were housed in cages in a 12 hr:12 hr light-dark cycle condition, with access to food and water *ad libitum*. After more than 10 days, they were placed in a condition of constant darkness when the data collection commenced. All the protocols for using animals in this study were approved by the Osaka Bioscience Institute Animal Research Committee. Piezoelectric sensor sheets (25 cm×15 cm) placed under the cages were used to measure the daily activity of the mice. The sensor sheets output voltage signals that are proportional to the pressure generated by the mice's activity. The signals were sampled at 100 Hz with 16-bit resolution after passing through the band-pass filter with 0.5–50 Hz. The signals were then digitized and stored on a computer. To construct locomotor activity data which corresponds to human locomotor activity data, we conducted the following processing of the measured sensor data. Firstly, we divided the sensor data into intervals (windows) of equal length *n*. In each window, we fit a linear function, which represents the trend in that window. Then, by subtracting the local trend in each window, the variance of the detrended sensor data was calculated. The time series of the variance obtained were regarded as the locomotor activity data of the mice.

### Analysis of locomotor activity data

To evaluate quantitatively locomotor activity patterns, we estimated the cumulative probability distribution *P*(*x*≥*a*) of durations *a* both of resting periods, where the activity counts are successively lower than a certain predefined threshold value, and of active periods, where the counts are successively higher than the threshold. The cumulative probability distributions were obtained by numerically integrating the estimated probability density function *p*(*x*) with a bin width of 1 min, as follows 
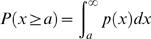
. Rescaled cumulative distributions 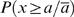 were also estimated from the rescaled probability density function, where both resting and active period durations are respectively rescaled by those of the individual means. In this case, the bin width is set to 0.1. Note that, in Figs. 2–5, we show the group averaged cumulative distributions with wider bins for the purpose of illustration, while the fitting procedure below was conducted using values in bins of 1 min (for *P*(*x*≥*a*)) or 0.1 (for 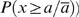).

We fitted a power law form *P*(*x*≥*a*) = *Aa* ^−*γ*^ to cumulative distributions of resting periods, and a stretched exponential functional form *P*(*x*≥*a*) = exp(−*αa* *^β^*) to those of active periods. For the objective selection of a fitting scale range, we examined the influence of the choice of threshold values (Fig. 3) and data resolutions (Fig. 4). Based on the robustness observed for cumulative distributions of resting period durations up to about 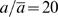, especially against the data resolution (Figs. 4**a, d**), we set the fitting range to 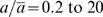. On the other hand, the cumulative distributions of active period durations show such robustness above 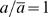, therefore, we fitted them to a range 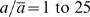.

The fitting parameters were estimated based on the minimum of the chi-square statistic *χ* ^2^ [21], Akaike Information Criterion (AIC) [22] and Bayesian Information Criterion (BIC) [23] (Appendix S1; Tables S1, Table S2, Table S3 and Table S4). Although the choice of a power-law form for resting period distributions, based on the high linearity in the log-log plots (see Figs. 2–5), is straightforward, there can be multiple choices of the model for active period distributions. We therefore also tried to fit an integrated (cumulative) distribution of the exact waiting-time probability distribution derived from the Barabási's model with two tasks [24] and a power-law distribution plus exponential cut-off, in addition to the stretched exponential form, to active period durations (Appendix S1; Table S5, Table S6 and Table S7; Figure S1). These alternative models also fit active period distributions quite well, although the fit for the stretched exponential form is slightly better in terms of *χ* ^2^ and AIC. We therefore used the stretched exponential form for active period distributions in the present study because this is a simple, as well as parsimonious, model.

### Statistics

The Kruskal-Wallis test was used to compare the mean parameter values among groups, followed by Steel-Dwass multiple comparisons. The *p*<0.05 was considered significant.

## Supporting Information

Appendix S1 Supporting information text.
(0.13 MB PDF)

Table S1 Goodness of fit of the power-law model for rescaled cumulative distributions of resting periods with various threshold values.
(0.07 MB PDF)

Table S2 Goodness of fit of the stretched exponential model for rescaled cumulative distributions of active periods with various threshold values.
(0.07 MB PDF)

Table S3 Goodness of fit of power-law model for rescaled cumulative distributions of resting periods with different data resolutions.
(0.06 MB PDF)

Table S4 Goodness of fit of stretched exponential model for rescaled cumulative distributions of active periods with different data resolutions.
(0.06 MB PDF)

Table S5 Goodness of fit of stretched exponential model for rescaled cumulative distributions of active periods.
(0.05 MB PDF)

Table S6 Goodness of fit of cumulative Vazquez' Eq. 8 for rescaled cumulative distributions of active periods.
(0.10 MB PDF)

Table S7 Goodness of fit of cumulative power-law with exponential cut-off for rescaled cumulative distributions of active periods.
(0.05 MB PDF)

Figure S1 Comparison of alternative fitting models for rescaled cumulative distribution of active periods.
(0.17 MB PDF)
