# Supplementary material for: Of Mice and Men — Universality and Breakdown of Behavioral Organization
Source: PLoS One. 2008 Apr 30;3(4):e2050. doi: 10.1371/journal.pone.0002050 (PMC2323110; doi:10.1371/journal.pone.0002050)
Supplement: Appendix S1 — Supporting information text. (0.13 MB PDF) [file pone.0002050.s001.pdf]

Supplementary Information to "Of Mice and Men - Universality and Breakdown of Behavioral Organization" by Nakamura et al.

We fitted a power law model $P(x) \sim x^{-\gamma}$ to the cumulative distributions of resting periods, and a stretched exponential model $P(x) \sim e^{-\alpha x^{\beta}}$ to the cumulative distributions of active periods. In order to evaluate the goodness of fit for our fitting models quantitatively, we calculated the following four statistical measures (for the cumulative distributions shown in Figs. 3–4):

1. Residual: $Err = \sum_i^N (P_{data}(i) - P_{fit}(i))^2$,
2. Chi-square statistic: $\chi^2 = \sum_i^N (P_{data}(i) - P_{fit}(i))^2 / (P_{data}(i) + P_{fit}(i))$,
3. Akaike information criterion: $AIC = -2Ł + 2k$,
4. Schwarz's Bayesian information criterion: $BIC = -2Ł + k\ln(N)$,

where P_{data} and P_{fit} denote the observed and approximated cumulative distributions, respectively. i represents the index number of a histogram bin, N is data number for fitting and $Ł$ and k are the likelihood function and the number of parameters in the model.

In addition, we also provided the goodness of fit for alternative fitting models (Barabasi's model and power-law distribution with exponential cut-off).

1. Comparison of goodness of fit for Fig. 3: Dependency on threshold values

In Fig. 3, we show the influence of the choice of threshold values for cumulative distributions of resting and active periods. Then, we provide the invariance of the results with the threshold values used. Tables S1–S2 provide the goodness of fit for rescaled cumulative distributions of both resting and active periods respectively, with different threshold values ranging from 0.6 to 1.6 times the overall non-zero activity counts in both adolescents and wild-type mice. In this case, the fitting range is set to $a/\bar{a} = [0.2, 20]$ for resting periods and $a/\bar{a} = [1, 25]$ for active periods.

All measures of goodness of fit mark the minimum values around threshold value $= 1.0$.

2. Comparison of goodness of fit for Fig. 4: Dependency on data resolutions

We also examined the influence of data resolutions, and show the results in Fig 4. Tables S3–S4 provide the goodness of fit of the cumulative distributions for different

data resolutions. We set the fitting range to $a/\bar{a} = [0.2, 20]$ for resting periods and $a/\bar{a} = [1, 25]$ for active periods. In the longer scales, the distributions of resting periods gradually converge to the overall slope when increasing the data resolution. Indeed, all measures of goodness of fit show decreasing tendencies with increasing data resolutions.

3. Comparison of goodness of fit for other fitting models

Other than the stretched exponential used in the main text and evaluated above, there can be multiple other choices of formal model fitted to the active period distributions. We therefore also attempted to fit (1) an integrated (cumulative) distribution such as that obtained analytically for the waiting-time probability distribution in the Barabasi's model with two tasks, Eq. 8 in [Vazquez A. et al, Phys. Rev. Lett. 95, 248701 2005], and (2) a power-law distribution with an exponential cut-off, such as that obtained analytically for the waiting-time probability distribution with two priority levels [Abate J. et al, Queuing Systems 25, 173-233, 1997].

1. Stretched exponential: $P(x) = e^{-\alpha x^{\beta}}$

2. Cumulative power-law with exponential cut-off : $P(x) = C\int_x^{\infty} t^{-\gamma} e^{-\alpha t} dt$

3. Cumulative model (Vazquez' Eq. 8) :

$$P(x) = C\int_x^{\infty} \frac{1-p^2}{4p}\left[\left(\frac{1+p}{2}\right)^{t-1} - \left(\frac{1-p}{2}\right)^{t-1}\right]\frac{1}{t-1}\,dt (t>1)$$

These alternative models also fit active period distributions quite well (Tables S5–S7 and Fig. S1), although the fit for the stretched exponential form is slightly better in terms of four measures.
