# Supplementary material for: Of Mice and Men — Universality and Breakdown of Behavioral Organization
Source: PLoS One. 2008 Apr 30;3(4):e2050. doi: 10.1371/journal.pone.0002050 (PMC2323110; doi:10.1371/journal.pone.0002050)
Supplement: Table S1 — Goodness of fit of the power-law model for rescaled cumulative distributions of resting periods with various threshold values. (0.07 MB PDF) [file pone.0002050.s002.pdf]

Table S1. Goodness of fit of the power-law model: $P(x) = Ax^{-\gamma}$ for rescaled cumulative distributions of resting periods with various threshold values.

	Threshold values	$Err \times 10^{-5}$	$\chi^2 \times 10^{-3}$	AIC	BIC
Adolescents	0.6	37.4 ± 38.4	39.8 ± 40.2	-1085 ± 194	-1078 ± 194
	0.8	21.6 ± 15.5	23.9 ± 17.5	-1192 ± 227	-1185 ± 227
	1	13.4 ± 7.65	18.3 ± 10.1	-1240 ± 140	-1233 ± 140
	1.2	10.6 ± 5.05	14.6 ± 5.66	-1272 ± 113	-1265 ± 113
	1.4	12.4 ± 7.55	13.0 ± 5.19	-1253 ± 129	-1247 ± 129
	1.6	25.1 ± 27.7	18.2 ± 9.83	-1136 ± 141	-1129 ± 141
WT Mice	0.6	2.12 ± 0.86	3.78 ± 3.17	-1926 ± 136	-1919 ± 136
	0.8	2.34 ± 1.25	2.62 ± 1.80	-1909 ± 141	-1902 ± 141
	1	2.18 ± 0.97	2.35 ± 1.55	-1910 ± 96	-1903 ± 96
	1.2	2.81 ± 1.04	2.60 ± 1.66	-1845 ± 88	-1838 ± 88
	1.4	3.29 ± 1.52	2.72 ± 1.48	-1813 ± 103	-1806 ± 103
	1.6	3.97 ± 1.86	2.95 ± 1.57	-1771 ± 112	-1764 ± 112
