# Supplementary material for: Of Mice and Men — Universality and Breakdown of Behavioral Organization
Source: PLoS One. 2008 Apr 30;3(4):e2050. doi: 10.1371/journal.pone.0002050 (PMC2323110; doi:10.1371/journal.pone.0002050)
Supplement: Table S2 — Goodness of fit of the stretched exponential model for rescaled cumulative distributions of active periods with various threshold values. (0.07 MB PDF) [file pone.0002050.s003.pdf]

Table S2. Goodness of fit of the stretched exponential model: $P(x) = e^{-\alpha x^{\beta}}$ for rescaled cumulative distributions of active periods with various threshold values.

	Threshold values	$Err \times 10^{-5}$	$\chi^2 \times 10^{-3}$	AIC	BIC
Adolescents	0.6	3.54 ± 2.58	13.4 ± 6.23	-1843 ± 193	-1836 ± 193
	0.8	1.81 ± 1.15	10.2 ± 4.92	-1980 ± 150	-1973 ± 150
	1	1.62 ± 0.82	8.21 ± 4.25	-1991 ± 123	-1984 ± 123
	1.2	1.47 ± 0.65	7.39 ± 3.08	-2004 ± 95	-1997 ± 95
	1.4	1.98 ± 1.08	8.22 ± 4.40	-1945 ± 124	-1938 ± 124
	1.6	3.17 ± 2.15	10.4 ± 5.74	-1844 ± 142	-1837 ± 142
WT Mice	0.6	2.24 ± 0.76	3.08 ± 1.80	-1907 ± 124	-1900 ± 124
	0.8	2.43 ± 1.29	2.55 ± 1.78	-1901 ± 146	-1894 ± 146
	1	2.27 ± 1.13	2.41 ± 1.58	-1904 ± 105	-1897 ± 105
	1.2	2.75 ± 0.95	2.56 ± 1.64	-1849 ± 84	-1842 ± 84
	1.4	3.53 ± 1.81	2.83 ± 1.57	-1802 ± 116	-1795 ± 116
	1.6	3.89 ± 1.75	2.90 ± 1.53	-1774 ± 108	-1767 ± 108
