# Supplementary material for: Of Mice and Men — Universality and Breakdown of Behavioral Organization
Source: PLoS One. 2008 Apr 30;3(4):e2050. doi: 10.1371/journal.pone.0002050 (PMC2323110; doi:10.1371/journal.pone.0002050)
Supplement: Table S3 — Goodness of fit of power-law model for rescaled cumulative distributions of resting periods with different data resolutions. (0.06 MB PDF) [file pone.0002050.s004.pdf]

Table S3. Goodness of fit of power-law model: $P(x) = Ax^{-\gamma}$ for rescaled cumulative distributions of resting periods with different data resolutions.

	Resolution	$Err \times 10^{-5}$	$\chi^2 \times 10^{-3}$	AIC	BIC
Adolescents	10s	6.67 ± 6.01	4.33 ± 2.68	-1413 ± 184	-1407 ± 184
	30s	8.83 ± 6.03	9.67 ± 4.44	-1334 ± 152	-1327 ± 152
	60s	13.4 ± 7.65	18.3 ± 10.1	-1240 ± 140	-1233 ± 140
	90s	22.8 ± 14.9	25.3 ± 13.2	-1141 ± 147	-1135 ± 147
	120s	34.4 ± 21.9	31.9 ± 14.1	-1053 ± 132	-1046 ± 132
WT Mice	0.05s	2.82 ± 1.32	2.23 ± 0.64	-1853 ± 113	-1846 ± 113
	0.1s	2.22 ± 1.03	2.31 ± 1.54	-1907 ± 100	-1900 ± 100
	0.5s	1.90 ± 1.39	2.90 ± 1.48	-1964 ± 131	-1957 ± 131
	1s	1.41 ± 0.68	2.70 ± 1.33	-2024 ± 123	-2017 ± 123
	5s	1.01 ± 0.56	4.39 ± 2.30	-2101 ± 109	-2094 ± 109
	10s	1.48 ± 0.73	5.55 ± 2.57	-2007 ± 109	-2000 ± 109
