# Supplementary material for: Of Mice and Men — Universality and Breakdown of Behavioral Organization
Source: PLoS One. 2008 Apr 30;3(4):e2050. doi: 10.1371/journal.pone.0002050 (PMC2323110; doi:10.1371/journal.pone.0002050)
Supplement: Table S4 — Goodness of fit of stretched exponential model for rescaled cumulative distributions of active periods with different data resolutions. (0.06 MB PDF) [file pone.0002050.s005.pdf]

Table S4. Goodness of fit of stretched exponential model: $P(x)=e^{-\alpha x^{\beta}}$ for rescaled cumulative distributions of active periods with different data resolutions.

	Resolution	$Err \times 10^{-5}$	$\chi^2 \times 10^{-3}$	AIC	BIC
Adolescents	10s	1.43 ± 0.65	3.19 ± 1.56	-2015 ± 105	-2008 ± 105
	30s	1.29 ± 0.76	4.92 ± 2.70	-2048 ± 122	-2041 ± 122
	60s	1.62 ± 0.82	8.21 ± 4.25	-1991 ± 123	-1984 ± 123
	90s	1.75 ± 1.01	10.7 ± 4.58	-1978 ± 127	-1971 ± 127
	120s	2.30 ± 1.20	13.5 ± 7.08	-1911 ± 129	-1904 ± 129
WT Mice	0.05s	3.04 ± 1.26	2.20 ± 0.62	-1831 ± 104	-1824 ± 104
	0.1s	2.27 ± 1.13	2.41 ± 1.58	-1904 ± 105	-1897 ± 105
	0.5s	1.94 ± 1.36	2.69 ± 1.49	-1954 ± 120	-1947 ± 120
	1s	1.34 ± 0.73	2.73 ± 1.31	-2042 ± 136	-2035 ± 136
	5s	1.03 ± 0.55	4.58 ± 2.15	-2094 ± 102	-2087 ± 102
	10s	1.49 ± 0.72	6.22 ± 3.13	-2004 ± 105	-1997 ± 105
