# Supplementary material for: Of Mice and Men — Universality and Breakdown of Behavioral Organization
Source: PLoS One. 2008 Apr 30;3(4):e2050. doi: 10.1371/journal.pone.0002050 (PMC2323110; doi:10.1371/journal.pone.0002050)
Supplement: Table S5 — Goodness of fit of stretched exponential model for rescaled cumulative distributions of active periods. (0.05 MB PDF) [file pone.0002050.s006.pdf]

Table S5. Goodness of fit of stretched exponential model: $P(x) = e^{-\alpha x^{\beta}}$ for rescaled cumulative distributions of active periods.

	$\bar{\alpha}$	$\bar{\beta}$	$Err \times 10^{-5}$	$\chi^2 \times 10^{-3}$	AIC	BIC
Adults	1.37	0.59	2.59 ± 1.49	9.68 ± 4.38	-1887 ± 143	-1880 ± 143
Adolescents	1.43	0.54	1.62 ± 0.82	8.21 ± 4.25	-1991 ± 123	-1984 ± 123
Depression	1.52	0.49	1.47 ± 1.25	4.09 ± 2.04	-2038 ± 154	-2031 ± 154
WT Mice	1.46	0.59	2.27 ± 1.13	2.41 ± 1.58	-1904 ± 105	-1897 ± 105
Per2 Mice	1.51	0.56	3.84 ± 1.94	3.72 ± 1.94	-1784 ± 129	-1778 ± 129
