# Supplementary material for: Of Mice and Men — Universality and Breakdown of Behavioral Organization
Source: PLoS One. 2008 Apr 30;3(4):e2050. doi: 10.1371/journal.pone.0002050 (PMC2323110; doi:10.1371/journal.pone.0002050)
Supplement: Table S6 — Goodness of fit of cumulative Vazquez' Eq. 8 for rescaled cumulative distributions of active periods. (0.10 MB PDF) [file pone.0002050.s007.pdf]

Table S6. Goodness of fit of cumulative Vazquez' Eq. 8: $P(x) = C\int_x^{\infty} \frac{1-p^2}{4p}\left[\left(\frac{1+p}{2}\right)^{t-1} - \left(\frac{1-p}{2}\right)^{t-1}\right]\frac{1}{t-1}dt$ **for rescaled cumulative distributions of active periods.**

	$\bar{p}$	$\bar{C}$	$Err \times 10^{-5}$	$\chi^2 \times 10^{-3}$	AIC	BIC
Adults	0.95	38.03	5.61 ± 4.87	12.8 ± 5.04	-1719 ± 160	-1712 ± 160
Adolescents	0.96	28.15	2.25 ± 1.17	8.24 ± 3.42	-1915 ± 128	-1908 ± 128
Depression	0.96	20.56	3.54 ± 2.20	6.25 ± 3.23	-1834 ± 198	-1827 ± 198
WT Mice	0.95	30.62	7.48 ± 5.18	7.62 ± 4.82	-1637 ± 143	-1630 ± 143
Per2 Mice	0.95	25.96	7.18 ± 2.67	7.86 ± 3.15	-1617 ± 77	-1610 ± 77
