# Supplementary material for: Of Mice and Men — Universality and Breakdown of Behavioral Organization
Source: PLoS One. 2008 Apr 30;3(4):e2050. doi: 10.1371/journal.pone.0002050 (PMC2323110; doi:10.1371/journal.pone.0002050)
Supplement: Table S7 — Goodness of fit of cumulative power-law with exponential cut-off for rescaled cumulative distributions of active periods. (0.05 MB PDF) [file pone.0002050.s008.pdf]

Table S7. Goodness of fit of cumulative power-law with exponential cut-off: $P(x)=C\int_x^\infty t^{-\gamma}e^{-\alpha t}dt$ **for rescaled cumulative distributions of active periods.**

	$\bar{\gamma}$	$\bar{\alpha}$	$Err \times 10^{-5}$	$\chi^2 \times 10^{-3}$	AIC	BIC
Adults	1.05	0.28	1.84 ± 0.79	10.0 ± 4.63	-1954 ± 114	-1947 ± 114
Adolescents	1.13	0.23	1.95 ± 1.01	8.21 ± 3.53	-1951 ± 132	-1944 ± 132
Depression	1.24	0.18	1.37 ± 0.86	5.39 ± 2.26	-2042 ± 140	-2035 ± 140
WT Mice	1.11	0.29	2.23 ± 1.29	3.84 ± 2.52	-1921 ± 136	-1914 ± 136
Per2 Mice	1.16	0.26	3.19 ± 2.39	5.43 ± 2.85	-1842 ± 142	-1835 ± 142
