# Supplementary material for: Of Mice and Men — Universality and Breakdown of Behavioral Organization
Source: PLoS One. 2008 Apr 30;3(4):e2050. doi: 10.1371/journal.pone.0002050 (PMC2323110; doi:10.1371/journal.pone.0002050)

Figure S1. Comparison of alternative fitting models for rescaled cumulative distribution of active periods. Vazquez' Eq. 8 model is shown in red, stretched exponential model in blue, and power-law with exponential cut-off, in green.

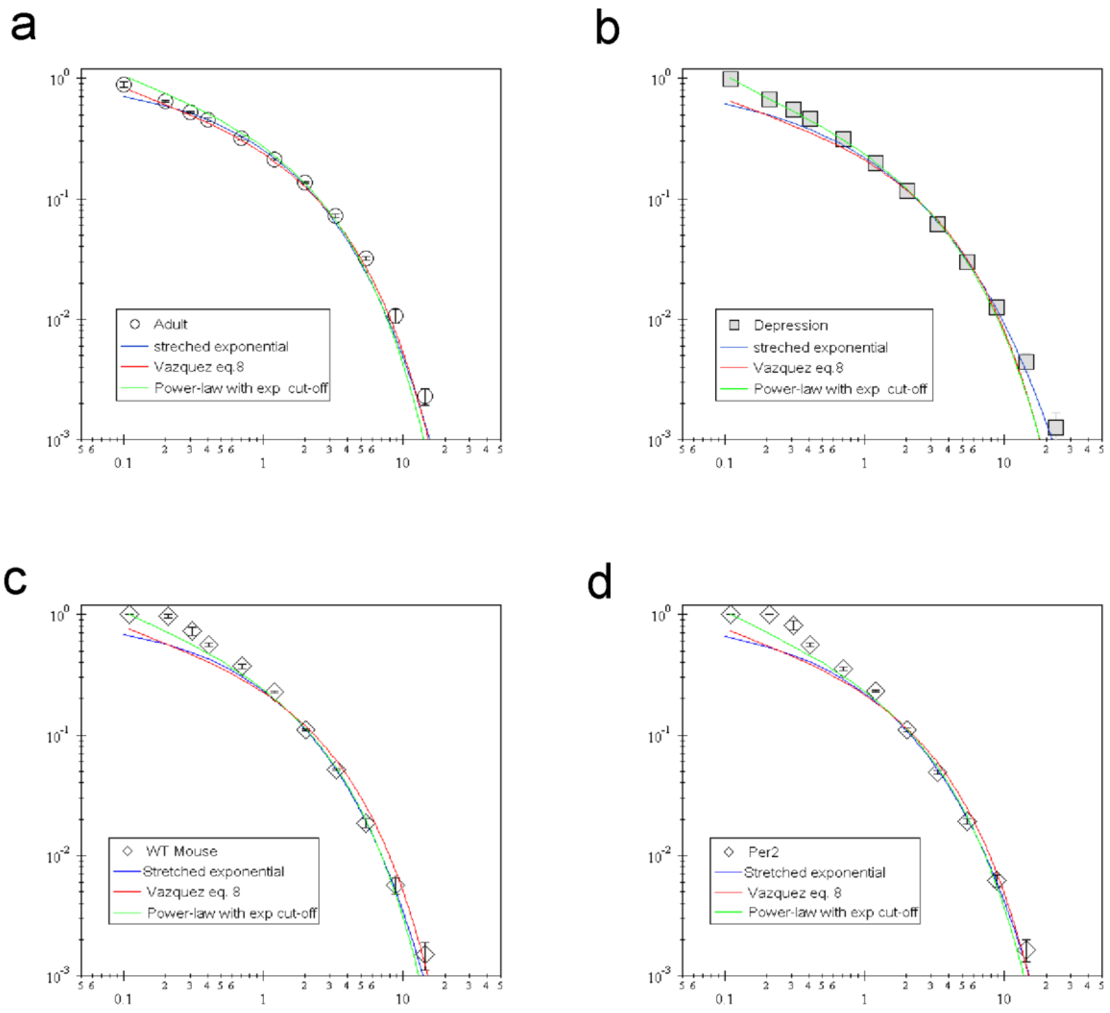

Supplement: Figure S1 — Comparison of alternative fitting models for rescaled cumulative distribution of active periods. (0.17 MB PDF) [file pone.0002050.s009.pdf]
